# Do Behavioral Interventions Increase the Intake of Biofortified Foods in School Lunch Meals? Evidence from a Field Experiment with Elementary School Children in Ethiopia

**DOI:** 10.1093/cdn/nzac008

**Published:** 2022-02-12

**Authors:** Julius J Okello, David R Just, Wellington Jogo, Norman Kwikiriza, Haile Tesfaye

**Affiliations:** International Potato Centre, Kampala, Uganda; The Charles H. Dyson School of Applied Economics and Management, Cornell University, Ithaca, NY, USA; International Potato Centre, Lilongwe, Malawi; International Potato Centre, Kampala, Uganda; International Potato Centre, Addis Ababa, Ethiopia

**Keywords:** school lunch meals, behavioral nudges, promotion, biofortified foods, acceptance, school children, Ethiopia

## Abstract

**Background:**

Many African countries are seeking to improve nutrition by introducing biofortified foods in school feeding programs. These programs are generally designed to create demand for biofortified foods both in and outside of school. Finding ways to encourage child acceptance of novel biofortified foods is key to the success of this strategy.

**Objectives:**

The aim was to assess effects of 2 behavioral interventions in promoting the consumption of biofortified foods as part of school lunch meals.

**Methods:**

The study is based on a field experiment involving 360 school-going children of in the third, fourth, and sixth grades. We tested if structured provision of information about the nutritional benefits of a biofortified food and its association with an aspirational figure influence its consumption when served alongside a favorite local food as part of school lunch meal. Six schools in Tigray, Ethiopia, were randomly selected to participate, with 4 participating in the Orange-Fleshed Sweetpotato (OFSP) Program. Of the 4 participating in the program, 2 were assigned to provide educational information about sweetpotato, whereas 2 presented the same information plus depictions of an aspirational figure (a famous local athlete) associated with the sweetpotato.

**Results:**

Provision of information on the nutritional benefits of biofortified food combined with an aspirational figure resulted in increased consumption of biofortified food by children. However, provision of the information alone did not detectably increase consumption. An analysis of trends over the course of the study revealed no discernable decay effect.

**Conclusions:**

Our results highlight the potential for relatively inexpensive behavioral interventions to increase acceptance of novel biofortified foods among children in a developing-country context. Larger studies with more varied interventions and larger numbers of participating schools could address several of the weaknesses in this study and establish more robust findings.

## Teaser

A field experiment with elementary school children in Ethiopia demonstrates that behavioral nudges combined with nutrition information increase intake of novel foods and provides a low-cost strategy for promoting biofortified foods.

## Introduction

In several countries around the globe, school feeding programs exist to achieve 3 broad goals: improved school enrollment, nutrition, and with both of these contributing to improved learning (1). Increasingly in several African countries, projects are tapping into this opportunity to introduce nutritious biofortified foods into the school lunch programs. In sub-Saharan Africa, such initiatives have been implemented in Ethiopia, Nigeria, Ghana, Tanzania, Rwanda, South Africa, and currently in Uganda (2–4). Malnutrition is a major public health problem in Ethiopia, where our study takes place. At the national level, 37% of children under 5 y are stunted, 7% are wasted, and 21% are underweight (5). However, the levels of malnutrition vary markedly across the different regions of Ethiopia. The Tigray region in northern Ethiopia is among the worst affected regions. The prevalence of stunting among children under 5 y of age in Tigray was approximately 49%, which is above the national average and is the worst in the country (5). Prevalences of wasting and underweight for the region were estimated at 9% and 30%, respectively, making Tigray a malnutrition hotspot.

Biofortified foods have proven effective in the fight against micronutrient deficiency among vulnerable populations (6–8). For instance, orange-fleshed sweetpotato (OFSP) has been shown to be effective in combating vitamin A deficiency among young children, pregnant women, and breastfeeding mothers. Regular consumption of a modest amount of just 125 g/d of OFSP is sufficient to meet the vitamin A requirements of early-age school children (age 5–10 y) (6, 9). In Uganda, there are plans to introduce OFSP into the primary school curriculum. The intention of targeting schools and school feeding programs is usually 2-fold. First, it is argued that, when young children are introduced to these nutritious foods, they will develop a liking for them and ultimately keep consuming them in the future as they grow up and become future parents (4, 6). Second, school children are targeted with the expectation that they will be ambassadors of the biofortified crops and act as agents of change in their families and communities (10). The Government of Ethiopia has emphasized the need for investments in interventions that promote consumption of nutrient-dense, biofortified crops to address malnutrition among women and children, especially among poor, remote rural communities. In line with government policy, the International Potato Center (CIP) and partners implemented an Irish Aid–funded project, “Scaling [up] potato and sweetpotato led intervention to improve nutrition and food security,” during the period 2013–2017. The project was aimed at scaling up production and consumption of nutritious OFSP varieties by smallholder farmers in the Tigray and Southern Nation Nationalities People's Region (SNNPR) of Ethiopia.

Malnutrition is prevalent particularly among school-age children in Ethiopia. Although large-scale studies are lacking, several small-scale studies show that the prevalence of stunting among school-age children ranges from 30% to 40% (11–13). Accordingly, as part of the scaling-up strategy, the project targeted school-age children by integrating OFSP into the World Food Program (WFP)–led school lunch program through introduction of OFSP school gardens and OFSP meals in 10 selected schools in the Tigray region. School children participated in the hands-on establishment and management of school gardens for vine multiplication and dissemination to neighboring communities. Once the vines matured, children were given some to take to their parents to plant at home. Additionally, harvested OFSP roots were either steamed alone or added to popular local foods and served to the school children as part of the school lunch. In addition to learning the basics of sweetpotato agronomy through their school agriculture clubs, children were also educated on general nutrition and specific aspects of OFSP nutrition through school science clubs. By 2018, a total of 18,450 school children from the 10 selected schools were being fed an OFSP meal as part of school lunch meals.

Enrichment of crops with micronutrients alters some of their sensory and even physical characteristics (14–16). This has led to concerns about their acceptability among targeted populations. Evidence from studies conducted at household and market levels indicates that both adult and school-age children tend to like biofortified crops. In the case of OFSP, the deep-orange color of the flesh makes it particularly attractive to children (10, 17). Sensory tests involving school children found that the yellow color of biofortified cassava makes it especially attractive to elementary school children (17). In a similar sensory evaluation study involving iron-rich beans, with no color differences, the authors found no statistical difference in acceptance/liking of the biofortified beans over the popular local variety (18). The acceptance of biofortified foods served as part of the school meal is not well understood. Given the presence of a popular culturally acceptable alternative, will school children consume foods made from biofortified crops? Lagerkvist et al. (14) report instances of school children discarding OFSP meals that had been introduced as part of the school lunch while consuming all the familiar local foods. De Groote et al. (16) found that mixing biofortified food into locally cooked popular foods made the food less acceptable. Talsma et al. (17) reported that biofortified yellow-fleshed cassava varieties were perceived to be inferior to the white types by consumers in Kenya.

The promotion of biofortified crops has often been accompanied by promotional campaign instruments (e.g., billboards and signs), branded materials (e.g., shirts, caps, umbrellas, and women's wraps), field-day events, and local FM radio broadcasts (19, 20). These promotion activities target the whole population—that is, both school-age and non–school-age individuals. In this study, we tested a behavioral strategy that has been used successfully in Western countries to promote the consumption of nutritious healthy foods in a school environment (21). Behavioral interventions have shown significant promise in providing children the motivation to both select and consume healthy foods (22, 23). These interventions use simple behavioral tools found in the fields of social psychology, behavioral economics, or marketing. There is a relatively large body of literature examining the potential to use behavioral interventions to influence the diets of school children (24). However, the vast majority of these studies have centered around the use of behavioral interventions in American and European schools and the public sector (23–28). More than 20% of schools in the United States reported using such interventions as part of what has been called the Smarter Lunchrooms Program (SLP) (24, 29). The principles of the SLP are deeply rooted in the social psychology literature and have only been applied to studies of food-consumption behavior in the relatively recent past. For example, Turnwald et al. (30) found that using attractive names for targeted foods can lead to an increase in overall selection and consumption. Wrapping a salad bar with images of popular puppet characters dramatically increased the selection of salad by public school students (23). In an experiment conducted in 5 European countries, Gwozdz et al. (27) found that stamping children with a smiley face increased the choice of vegetable salads among primary school children. Several have studied the impact of inclusion of healthy foods in the meal by default (rather than on request) on consumption (31, 32). Such behavioral tools can work to motivate children to select and eat new foods and have the advantage of being both relatively effective and relatively low cost (24).

Interventions that appear too heavy-handed often backfire. One recent study conducted in West Africa found that overemphasizing the health benefits of OFSP was not effective in influencing acceptance of OFSP meals among school children in a school lunch setting (3). It is unclear how important behavioral tools will be in an environment where most children experience food insecurity. It could be that if children face food insecurity, they might already willingly eat whatever additional food might be placed before them. Can behavioral interventions still work in such an environment? There is some reason to believe that behavioral interventions are more effective among those facing scarcity generally (33). If children are willing to eat anything placed in front of them, whether familiar or not, then expenditures on influencing behavior through behavioral interventions may be superfluous. However, if behavioral interventions can influence how school children behave in such an environment, this would demonstrate the importance of seriously considering behavioral responses in designing strategies for promoting nutritious biofortified foods through schools. We tested the effectiveness of these behavioral interventions using a field experiment involving public elementary school children in the Tigray region of Ethiopia. We specifically tested the effect of information on the benefits of OFSP and association of OFSP with a celebrity (i.e., aspirational role model) on school children's acceptance and consumption of OFSP as part of a school meal.

This paper seeks to answer 2 main research questions. The first research question is, Does provision of information about the benefits of OFSP to school children increase their acceptance and consumption in the presence of local favorites? The second research question is, Does association of OFSP with an aspirational figure and its portrayal as being novel, associated with achievement, and an “academic performance booster” increase its consumption relative to popular local food? We define an aspirational figure as a person or character who is well known among the population and associated with positive attributes one would wish to emulate.

## Methods

### Study design

The study was conducted as a field experiment with 3 conditions. Two of the conditions exposed students to the WFP curriculum on OFSP and information about health and production benefits of OFSP, including cooking and gardening demonstrations. The first condition (T1) exposed children to the OFSP curriculum and meals alone. The second condition (T2) exposed children to the OFSP curriculum, and also associated OFSP with an aspirational figure. A control condition (T3) introduced the OFSP without any information or messaging. This design was intended to allow us to separately identify the effects of the curriculum and the marginal effect of adding messaging associated with the aspirational figure. Selecting an aspirational figure in the context of rural Ethiopia is a challenge. In the United States and Europe, TV characters, cartoons, puppets, celebrities, and smiley faces have all been used to influence food choice. Unlike Western countries, cartoon characters or other child-targeted programming are not universally recognized cultural references. In rural Ethiopia, exposure to pop culture, TV characters, or emojis is not common. Informal discussions with children in the region (not at participating schools) suggested that athletes can play a similar role. Athletes are associated with positive attributes such as strength, health, and fitness, and often have recognizable names and images. An image of Genzebe Dibaba was selected based on the general name and image recognition. Genzebe Dibaba is Ethiopian and is a current world record holder for several events. The image of Dibaba was displayed and linked to 3 attributes/aspects that the athlete represents, namely, “Strength,” “Power,” and “Success.” Images of a child eating the OFSP would appear superimposed on that of Dibaba, and teachers would refer to the image when instructing on OFSP.

### Allocation of study groups and sampling

The field experiment was conducted as a randomized controlled trial with treatments implemented at the school level, but using students as the unit of measurement. Given the pilot nature of this study caused by budget limitations, we were limited in the number of schools we could use in the study. Hence, only a total of 6 schools in Tigray region, Ethiopia, were included. Children in all schools in the study received a hot meal made from wheat, maize, and beans on every school day as part of the WFP school lunch program (i.e., Monday to Friday) (34, 35). We randomly selected 4 schools from a list of schools that were participating in the WFP school lunch program and had hosted OFSP school garden and cooking demos. This included Endamaino, Hatset, Adibire, and Maiweyne. A random-number generator was used to assign 2 schools to T1 and 2 schools to T2. The remaining 2 schools were randomly selected from a list of schools participating in the WFP program that were slated to participate but had hitherto not participated in the school gardening and cooking demonstrations involving OFSP, but had not yet implemented the program. Hence, subjects in this condition, T3, had not learned (and therefore had no information) about the benefits of OFSP. The 2 schools selected were Burka and Golgol Nael. Given the limited number of schools we could reach, and the limited number of similar studies with reported results, we conducted a preliminary power analysis using the simr package in R statistical software (R Foundation for Statistical Computing). [This analysis presumed a linear regression analysis comparing 2 treated with 2 control schools. Standard deviation of the percentage of OFSP consumed was drawn from reference 3. No similar studies in a developing country context targeting OFSP or similar foods could be found to determine expected effect sizes. Hence, we drew from an (unpublished) experimental study in a large metropolitan US district that included starchy vegetables. Intra-school correlation had been found to be negligible in prior studies in sub-Saharan Africa (ranging from 0.04 to 0.13).] This analysis suggested we needed a sample of 45 students per school to obtain a power of 0.80. Given the uncertain nature of some of the inputs to this calculation, and the potential for absences, we selected a sample size of 60 per school.

In each participating school, 3 grades were randomly selected from among the 7 elementary grades, yielding second, third, and sixth grades. Finally, in each selected grade, 20 students were randomly selected from the class roll/register to take part in the study. This resulted in 60 subjects being observed repeatedly on each observation/study day (6 d in total) in each school, for a total of 120 students per treatment and 360 subjects overall, or 2160 potential child-day observations. This number could erode due to absences.

### Field protocol and data collection

In each of the schools, OFSP was served to all children as part of the WFP lunch on all study days. This meal included OFSP, injera, and a traditional sauce called shuro. Injera is a soft and spongy bread made from teff flour with a somewhat sour flavor. Injera is generally dipped in sauces when consumed. Shuro sauce is a warm blend of chickpea flour, spices, and vegetables commonly served with injera. The amounts of injera and shuro sauce served were standardized at 400 g. The amount of OFSP meal served was also standardized at 320 g. This exceeds the 250 g of OFSP required to provide the daily vitamin A requirement of a young child (6). Hence, each participant received a total of 750 g of food altogether. A full description of the treatment narrative is available in the **Supplemental Data**.

In each school, while 60 students were observed, all students would receive the OFSP meal. The subjects and their nonparticipating classmates were not informed of whom was being observed, and the same meals and dishes were served to all children in the school. The meal was served in a bowl with a sticker/label bearing the subject's name and grade. Researchers could discern which students were in the study based on subtle differences in the stickers on the bowls. These stickers also provided identifiers that could be matched both to study enrollment information, consumption data across study days, as well as demographic data collected using a short module on the last day of the study. Children ate in the usual designated lunchroom/area. On each observation day, research assistants recorded, for each of the 60 participating students in the school, the weight of OFSP served and the weight of leftover OFSP (i.e., waste). In addition, the research assistants used the quarter-plate waste method (23, 36–38) to estimate the remaining injera and other waste. The module used to collect demographic data included subject's age, gender, number of siblings, whether a parent is employed (proxy for income status), and if the parent has ever grown OFSP. Observations were made on the same students every Tuesday and Friday over a period of 3 wk. On days not included in the study, students would be served meals that did not include OFSP, and food consumption was not measured. These days were included to prevent the food from becoming monotonous.

This study was conducted in accordance with the guidelines laid down in the Declaration of Helsinki. It was implemented as part of a project co-implemented by CIP and the Ethiopia Bureau of Education under a Memorandum of Understanding 11011–000-00-BoE Tigray-01, and all procedures involving study participants were officially approved by the respective school authorities (reference: HATSET 2/1–49/07 dated 22 December 2015 for Hatset Elementary School, for example). Additionally, the consent of parents to involve school children in the study was obtained for the children who were assessed.

### Statistical analysis

While random assignment is useful in helping to identify causal effects of our interventions, children could only be assigned to treatment at the school level, creating the possibility of heterogeneous samples and within-treatment correlation. We controlled for observable heterogeneity between samples by using regression analysis to estimate the effect of our intervention. We made estimations using the following equation:

(1) $$\begin{eqnarray*}
{y_{it}} = {\beta _o} + \sum\nolimits_{j \in T} {{\beta _j}{D_{ij}} + } \sum\nolimits_k {{\gamma _k}{X_{ik}}} + \sum\nolimits_{t = 2}^6 {{\alpha _t}Da{y_{it}} + {\varepsilon _{it}}}
\end{eqnarray*}$$

where *y_it_* is the amount of OFSP consumed in grams f)or individual *i* on day *t*, *D_ij_* is a dummy variable indicating if individual *i* was included in treatment *j*, *X_ik_* are a set of individual specific demographic variables, *Day_it_* are dummy variables indicating the day of observation, *ε_it_* is a disturbance term, and *β_j_*, *γ_k_*, and *α_t_* are coefficients to be estimated. Treatments include (control condition omitted to allow for a constant term) the following: *J* = (Information, Information + Aspirational). Sociodemographic controls are included for both age and gender.

We used 3 different methods to estimate the regression equations. Our preferred estimation uses a regression model with multilevel mixed-effects estimation (39). This conforms to our study design, with children nested within schools. Moreover, observations are left and right truncated, with approximately one-third of observations consuming all of the OFSP, and a small fraction consuming none. Thus, our primary model estimates Equation 1 using a mixed-effects Tobit regression (model 1) [see (40)].

To discern the robustness of the results, we also present 1 additional regression. We have repeated observations of subjects, which allows us to estimate a random-effects model with subject-specific random terms. Model 2 estimates the student-level random-effects Tobit model. Given the substantive number of censored truncated observations where children ate all of the OFSP offered, uncensored linear estimation would likely result in substantial bias. Model 3 estimates Equation 1 using a standard mixed-effects linear regression with students nested within schools. In both these regressions, the significance of the *β_j_* coefficients will yield the primary hypothesis tests of whether the treatment was effective in increasing consumption of OFSP.

The literature often suggests that behavioral treatments may have differential effects over time. Specifically, many have wondered if there might be relatively immediate novel effects, with effects decaying rather quickly. As an ad hoc test of this possibility, we ran 1 additional regression of the following form:

(2) $$\begin{eqnarray*}
{y_{it}} &=& {\beta _o} + \sum\nolimits_{j \in T} {{\beta _j}{D_{ij}}} + \sum\nolimits_{j \in T} \sum\nolimits_{t = 2}^6 {{\delta _{jt}}{D_{ij}}Da{y_{it}}}\\
&& +\, \sum\nolimits_k {{\gamma _k}{X_{ik}}} + \sum\nolimits_{t = 2}^6 {{\alpha _t}Da{y_{it}}} + {\varepsilon _{it}}
\end{eqnarray*}$$

We estimate this using the mixed-effects and random-effects Tobit models (models 1 and 2). Trends in consumption within treatment can be detected by determining if *δ_jt_* differs significantly over observation periods within treatment.

## Results

Approximately 53% of the study participants were male. The average ages of the students were 8, 10, and 12 for grades 2, 3, and 6 respectively. **Table 1** presents the demographic characteristics of the participants overall and by treatment. The ages of students within grade were fairly close across the treatments, although there is much less variation in age by grade in the Aspirational-figure treatment (T2). This treatment also displays a somewhat greater proportion of males that, for a given age, often consume more food per meal. Two other notable differences in the treatments are the somewhat greater familiarity with OFSP in the Information-only treatment (T1), 12.5% as opposed to 0–6% in all other treatments, and the relatively high percentage of households with mobile phone access in treatment T2. However, as expected, there was no knowledge of OFSP among subjects in the control group (T3), indicating that this group was not contaminated through spillover effects of the project's promotional activities.

**TABLE 1:** Demographic characteristics of the study participants^1^

Variable	Average (*n* = 360)	Control, T3 (*n* = 120)	Information, T1 (*n* = 120)	Information and Aspirational, T2 (*n* = 120)
Age (grade 6), y	12.28 (1.34)	12.10 (1.92)	12.60 (1.08)	12.15 (0.70)
Age (grade 3), y	9.68 (1.22)	9.55 (0.93)	10.48 (1.58)	9.00 (0.00)
Age (grade 2), y	8.34 (0.74)	8.175 (0.50)	8.85 (1.00)	8.00 (0.00)
Sex (*n* = 342)				
Male	52.7 (0.50)	48.33 (0.50)	50.83 (0.50)	59.17 (0.49)
Female	47.2 (0.50)	51.67 (0.50)	49.17 (0.50)	40.83 (0.49)
Number of siblings	4.04 (1.90)	3.88 (1.92)	3.70 (1.72)	4.53 (1.96)
Percentage with parent growing OFSP	5.56 (0.23)	0.00 (0.00)	12.50 (0.33)	4.17 (0.20)
Percentage with parents owning a phone	64.17 (0.48)	47.50 (0.50)	58.33 (0.50)	86.7 (0.34)

1 Values are percentages unless otherwise indicated. Values in parentheses are standard deviations (SD). OFSP, orange-fleshed sweetpotato.

**FIGURE 1:**
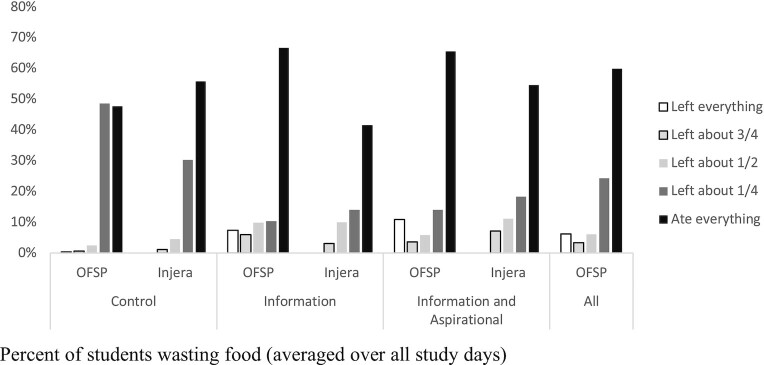
Percentage of students wasting food (averaged over all study days). This figure provides the proportion of students who left some food uneaten for each of the treatments. The horizontal axis is the food type (with OFSP representing the biofortified food while injera is the local popular staple Ethiopian food) for each treatment (control, information only, and information plus the nudge). The vertical axis presents the percentage of children who wasted some food by treatment type. OFSP, orange-fleshed sweetpotato.

**Figure 1** displays the distribution of plate waste observed among students in each treatment. The percentage wasted is represented in 4 bins based on the percentage of OFSP remaining on the plate. This allows us to compare the wasting rate to the more familiar food, injera. Here we observed that the model amount of OFSP left on the plate was approximately zero in all treatments except for the control, where it was approximately one-quarter. Alternatively, the model amount of injera wasted was approximately zero in all treatments. In treatments where less of the OFSP was wasted, there appears to be some small additional injera waste, suggesting perhaps some tradeoff between the 2 rather than purely additional consumption.

**Table 2** displays results of the regression equation estimation for both models. When using the nested mixed-effects Tobit model (model 1), information alone appears to have no detectable impact on OFSP consumed. This is also true using the random-effects Tobit model (model 2). The combination of information and an aspirational figure shows a positive and significant impact on OFSP consumption of approximately 27 g using both the mixed-effects and random-effects Tobit models. However, this effect is both smaller in magnitude and nonsignificant using the linear model. A test of the hypothesis that the coefficients for Information and Aspiration are equal fails to reject model 1 [χ(1)^2^ = 1.09, *p *= 0.297], but is rejected for model 2 [χ(1)^2^ = 16.59, *p *= 0.000]. This difference appears to be due to the relatively imprecise estimate of the effect on Information using the mixed-effects Tobit.

**TABLE 2:** Effect of promotional campaign on quantity (g) of OFSP consumption^1^

Variables	Model 1 (*n* = 2049), β (SE)	*P*	Model 2 (*n* = 2049), β (SE)	*P*
Control	Reference			
Information	–3.49 (29.23)	0.905	–5.35 (7.50)	0.476
Aspirational	25.87 (8.78)	0.003	26.34 (7.56)	0.000
Day 1	Reference			
Day 2	26.87 (11.44)	0.019	26.75 (6.22)	0.000
Day 3	111.10 (24.56)	0.000	111.32 (6.74)	0.000
Day 4	109.26 (24.42)	0.000	109.40 (6.76)	0.000
Day 5	91.15 (23.99)	0.000	91.53 (6.65)	0.000
Day 6	106.83 (22.38)	0.000	106.82 (6.72)	0.000
Female	Reference			
Male	22.69 (8.99)	0.012	29.00 (6.17)	0.000
Age, y	5.22 (2.56)	0.041	6.32 (1.58)	0.000
Constant	152.55 (26.07)	0.000	138.49 (17.09)	0.000

1 Model 1: mixed-effects Tobit with child embedded in school. Model 2: child-level random-effects Tobit. OFSP, orange-fleshed sweetpotato.

**Table 3** presents the results of both the mixed-effects and random-effects Tobit regressions estimating Equation 2. For the sake of readability, we present only the coefficients of interest for examining trends over the study period. Using the preferred model (model 1), there is no significant or discernable trend in OFSP consumption under either the Information-only or the Information and Aspirational-figure treatments. The coefficients for Information only are all positive, but individually nonsignificant. These coefficients are jointly significant (*P* = 0.000), suggesting that consumption increases in the Information treatment after the first day. The coefficients for the Information and Aspirational-figure treatment are also nonsignificant, with some positive and others negative. These coefficients are jointly significant (*P* = 0.000) but yield no discernable trend. When we examine using the random-effects Tobit model (model 2), the significance levels increase, reflecting the failure to capture of school-level effects, and some of the estimates change signs. In particular, now, all estimates associated with the Information-only treatment are positive, while those associated with the Aspirational-figure and Information are all negative, with 3 of these being significant. The coefficients for Information only are jointly significant (*P* = 0.014), suggesting that consumption increases after first introduction, although the estimated consumption effects are not monotonically increasing, and not suggestive that longer duration exposure would result in increasing acceptance. The coefficients for the Information plus Aspirational figure are also jointly significant (*P* = 0.000), suggesting that consumption decreases after the initial introduction. Again, this decline is not monotonic, and not particularly suggestive that longer exposure would decrease acceptance. In either case, the lack of a clear and discernable trend could easily be due to a lack of data with a relatively short-duration study.

**TABLE 3:** Trends of OFSP consumption (g) over the study duration by treatment groups^1^

Variables	Model 1 (*n* = 2049),^2^ β (SE)	*P*	Model 2 (*n* = 2049),^2^ β (SE)	*P*
Information only				
Day 1	Reference			
Day 2	16.253 (30.15)	0.590	15.02 (10.18)	0.140
Day 3	43.56 (23.45)	0.063	21.57 (10.10)	0.033
Day 4	40.82 (42.41)	0.336	9.98 (10.18)	0.327
Day 5	66.33 (49.75)	0.182	31.87 (10.17)	0.002
Day 6	15.01 (57.12)	0.793	2.33 (10.07)	0.817
Aspirational				
Day 1	Reference			
Day 2	–2.31 (18.18)	0.899	–7.82 (10.15)	0.441
Day 3	–52.76 (67.56)	0.435	–47.96 (10.18)	0.000
Day 4	22.54 (67.63)	0.739	–20.08 (10.16)	0.048
Day 5	7.30 (44.24)	0.869	–15.54 (10.23)	0.129
Day 6	–20.34 (37.82)	0.591	–34.19 (10.17)	0.001

1 Model 1: mixed-effects Tobit with child embedded in school. Model 2: child-level random-effects Tobit. OFSP, orange-fleshed sweetpotato

2 Adjusted for age, treatment, and sex. These coefficients are not reported for brevity.

## Discussion

This study assessed the effect of 2 behavioral interventions in promoting the consumption of biofortified foods as part of school lunch meals. There has been a rise in the number of developing-country projects targeting school lunch programs as vehicles for promoting biofortified food staples, often treating school children as agents of change. At the same time, the promotion of biofortified foods is normally accompanied by social behavior change communication to influence parents to plant and consume the nutritionally enhanced foods.

Using a field experiment involving elementary school children, we tested if structured provision of information about the nutritional benefits of a vitamin A–rich OFSP and association of OFSP with an aspirational figure (or role model) influences its consumption when served alongside a popular local food as part of a school lunch meal. We found that provision of information on nutritional benefits combined with associating OFSP with an aspirational figure increases its consumption, albeit with a small magnitude of effect (27 g, ∼8% of the serving). Provision of the information in the absence of the aspirational character did not detectably affect consumption. The small magnitude is likely due to the food situation in the study area at the time of study, during which a majority of households were facing food insecurity; hence, the likely tendency for the school children to finish everything placed before them. In such circumstances, it may be that behavioral tools are somewhat less effective than where deficiencies in the diet are prevalent despite relatively greater food security. Given the vast cultural differences across developing countries, substantial opportunities exist for unique and important interventions that are context and culture dependent. Finding successful behavioral interventions to support nutrition change will critically depend on designing and testing interventions with the local culture in mind.

Moreover, our findings do not detect any clear trend indicating a decay of the impact of the aspirational figure. More data would be needed to ensure that this result is robust. The issue of decay in behavioral results is an issue of importance as many have criticized behavioral interventions as being driven primarily by novelty effects that wear off relatively quickly (23).

Our study was hampered somewhat by the small number of schools we were able to include in the study. Creating cluster-randomized controlled trials with clustering at the school level could lead to more robust findings but would also require a substantially larger sample of participating schools. This underscores the need for larger and more varied interventions in developing-country schools. Larger studies will be able to more convincingly detect effects of behavioral interventions by more effectively accounting for school-level correlation of individual behavior. While we have intentionally focused on relatively homogenous populations to minimize school-level effects, this method is inherently limiting. Finally, appropriate analysis of group-randomized trials should reflect the number of randomized units, which, in our case, is 6. Due to the pilot nature of this study, the analysis was performed at the child level rather than the school level, accounting for clustering within school.

## Supplementary Material

nzac008_Supplemental_File

## Notes

Funding for this study was provided by the Irish Aid (CIP/Tigray and SNNPR/2013) through the International Potato Center.

Author disclosures: The authors report no conflicts of interest.

This research was undertaken as part of the CGIAR Research Program on Roots, Tubers and Bananas (RTB) and co-implemented by International Potato Center and the Ethiopia Bureau of Education under a Memorandum of Understanding 11011-000-00-BoE Tigray-01.

Abbreviations used: CIP, International Potato Center; OFSP, orange-fleshed sweetpotato; SLP, Smarter Lunchrooms Program; WFP, World Food Program.
