# Supplementary material for: Do Behavioral Interventions Increase the Intake of Biofortified Foods in School Lunch Meals? Evidence from a Field Experiment with Elementary School Children in Ethiopia
Source: Curr Dev Nutr. 2022 Feb 12;6(2):nzac008. doi: 10.1093/cdn/nzac008 (PMC8866104; doi:10.1093/cdn/nzac008)
Supplement: nzac008_Supplemental_File [file nzac008_supplemental_file.docx]

**Supplemental data**

**Supplementary information: Treatment 1 and 2 information narratives**

**Treatment 1**

Children in this group were reminded about the benefits of OFSP during brief talk in the school assembly by the Headteacher. The narrative provided was:

*“For the next few weeks, we are going to be having a school lunch for all the grades in this school. This lunch will have Injera and “Shuro” sauce which are your favorite traditional foods. In addition, the lunch this time will have a portion of orange-fleshed sweetpotato (OFSP) meal.*

*OFSP is good for your health because it provides energy and Vitamin A. The body needs vitamin A to prevent eye problems and childhood diseases such as diarrhea. Vitamin A is also very important for brain development and for good academic performance during school years.*

*During these weeks, we want each student to have his/her own bowl. So, each of you will have a bowl with your name written on it. The same amount of these foods will be served in these bowls. So, make sure you don’t take someone’s bowl. When you finish leave the bowl on the table. We have some people who will wash them for you. This will allow you more time to play before going back to class. Is that clear to everyone?"*

**Treatment 2**: In addition to receiving T1 information, the Headteacher provided a brief talk that relate OFSP with an inspiration character designed to capture students’ attention. The character used was Genzebe Dibaba, a popular widely known female track athlete and hero. The Headteacher explained, while showing a portrait of Dibaba, that eating OFSP would make one strong, have exceptional performance (i.e., successful) and be “cool”. The narrative provided was as follows:

*“For the next few weeks, we are going to be having a school lunch for all the grades in this school. This lunch will have Injera and “Shuro” sauce which is your favorite traditional food. In addition, the lunch this time will have a portion of OFSP meal. OFSP is good for your health because it provides energy and Vitamin A. The body needs vitamin A to prevent eye problems and childhood diseases such as diarrhea. Vitamin A is also very important for brain development and good academic performance during school years.*

*Eating OFSP results in healthy body and can make one strong, powerful and successful, just like our champion and hero Genzebe Dibaba the world champion runner. Eating OFSP is therefore “cool”.  I am sure you have heard of Dibaba [holding up a picture of Genzebe Dibaba] - our* *champion, star and hero."*

*During these weeks, we want each student to have his/her own bowl. So, each of you will have a bowl with his/her name written on it. The same amount of these foods will be served in these bowls as usual. So, make sure you don’t take someone’s bowl. When you finish leave the bowl on the table. We have some people who will wash them for you. This will allow you more time to play before going to class. Is that clear to everyone?"*

The class teachers of participating grades reinforced the treatment-specific message provided by the Headteacher just before lunch during each observation day to the students. For T2, the teacher concluded the remainder by pointing at the portrait of Dibaba mounted on the classroom wall and saying: Remember: “*Eating OFSP results in healthy body and can make one strong, powerful and successful, just like this champion and hero, Genzebe Dibaba, the world champion runner. Eating OFSP is therefore “cool”.*
